# Toward clinical translation of carbon nanomaterials in anticancer drug delivery: the need for standardisation

**DOI:** 10.3762/bjnano.16.144

**Published:** 2025-11-18

**Authors:** Michał Bartkowski, Francesco Calzaferri, Silvia Giordani

**Affiliations:** 1 School of Chemical Sciences, Dublin City University, Glasnevin, Dublin, Ireland; 2 Life Science Institute, Dublin City University, Glasnevin, Dublin, Ireland

**Keywords:** carbon nanomaterials (CNMs), carbon nanoparticles (CNPs), drug delivery systems (DDSs), nanocarriers, quality control (QC)

## Abstract

Carbon nanomaterials (CNMs), including graphene, carbon nanotubes, and carbon dots, have attracted considerable interest as nanocarriers for drug delivery due to their unique physicochemical properties. Their high surface area, biocompatibility, and modifiable surface chemistry make them highly attractive for a range of biomedical applications. However, concerns regarding toxicity and regulatory hurdles remain major barriers to clinical translation. Current research is therefore focused on standardizing CNM synthesis and characterisation methods, minimizing toxicity, and facilitating regulatory approval. Despite these challenges, CNMs hold substantial promise for enhancing therapeutic delivery, particularly in areas such as cancer treatment. This perspective highlights critical considerations in the development of CNM-based nanocarriers, spanning from initial design to clinical implementation.

## Introduction

### Nanomaterials

**Figure 1:**
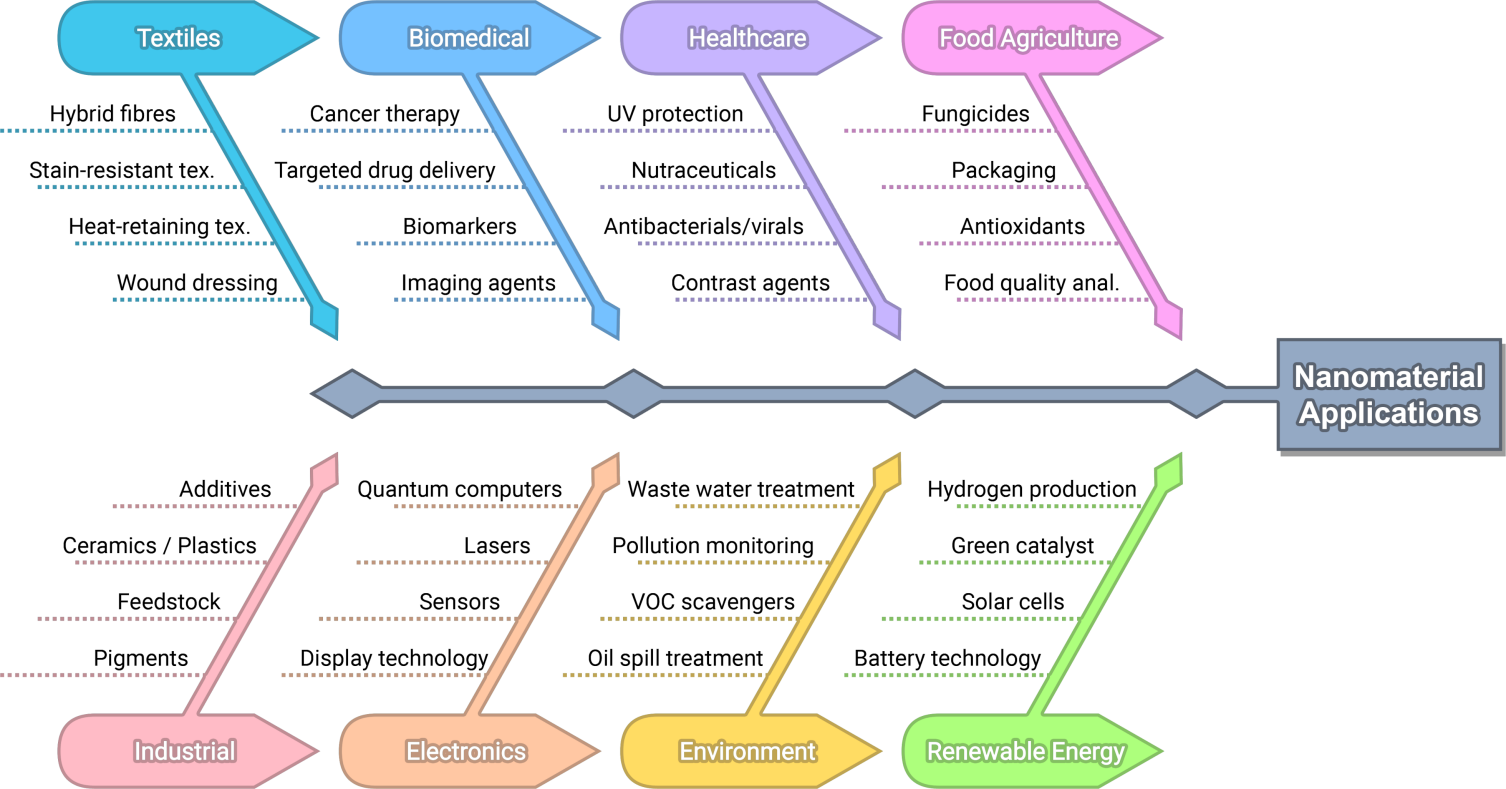
Overview of NM applications across various industries.

Nanomaterials (NMs) have an extensive array of various properties and applications across many industries, including the biomedical, health care, food/agriculture, industrial, environmental, electronic, and renewable energy sectors (Figure 1). NMs have seen use as antimicrobial agents [1], catalysts [2], bioimaging agents [3–6], magnetic particle imaging agents [7], nanofluids [8], antiviral agents [9], photothermal convertors [10], and in environmental remediation [11]. Topically, the biomedical applications of NMs have been extensive; including niche applications such as enabling nightvision; particular Yb(III) and Er(III) doped nanoparticles have been found to upconvert NIR light into visible light, thus enabling mice to visually perceive infrared light [12].

### Carbon nanomaterials as nanocarriers

Carbon nanomaterials (CNMs) have been largely developed as nanocarriers for drug delivery due to their biocompatibility, high surface area, tuneable physicochemical properties, and targeting capabilities [13–14]. However, CNMs also present a subset of challenges, including toxicity concerns, expensive and time-consuming production, and a lack of standardised methods for their characterisation. Nevertheless, growing research into CNM-based nanoformulations continues to address key challenges, underscoring their promise in enhancing therapeutic delivery; particularly in cancer treatment, where the global burden remains substantial.

This perspective uses oncology as a representative model for CNM nanocarriers, owing to cancer’s global health burden and the breadth of available studies [15–16]. Nevertheless, the underlying considerations, including synthesis, physicochemical and biological evaluation, and translational hurdles, extend broadly across therapeutic contexts. Regardless of indication, many of the key challenges in standardising CNM-based drug delivery systems remain largely the same.

### Using carbon nanocarrier to address the global burden of cancer

Cancer, a group of diseases characterised by uncontrolled cell growth and their potential to metastasise, poses a significant global health challenge. Numerous preventable factors contribute to cancer, including tobacco use, exposure to viruses, alcohol consumption, ultraviolet radiation (photocarcinogenesis), ionising radiation, poor diet and nutrition, sedentary behaviour, obesity, environmental contaminants, and certain occupations and pharmaceuticals [17]. Prevention, the most cost-effective and beneficial approach for the general population [18], is supported by strong evidence-based recommendations from organisations like the World Cancer Research Fund and the European Union [19].

**Figure 2:**
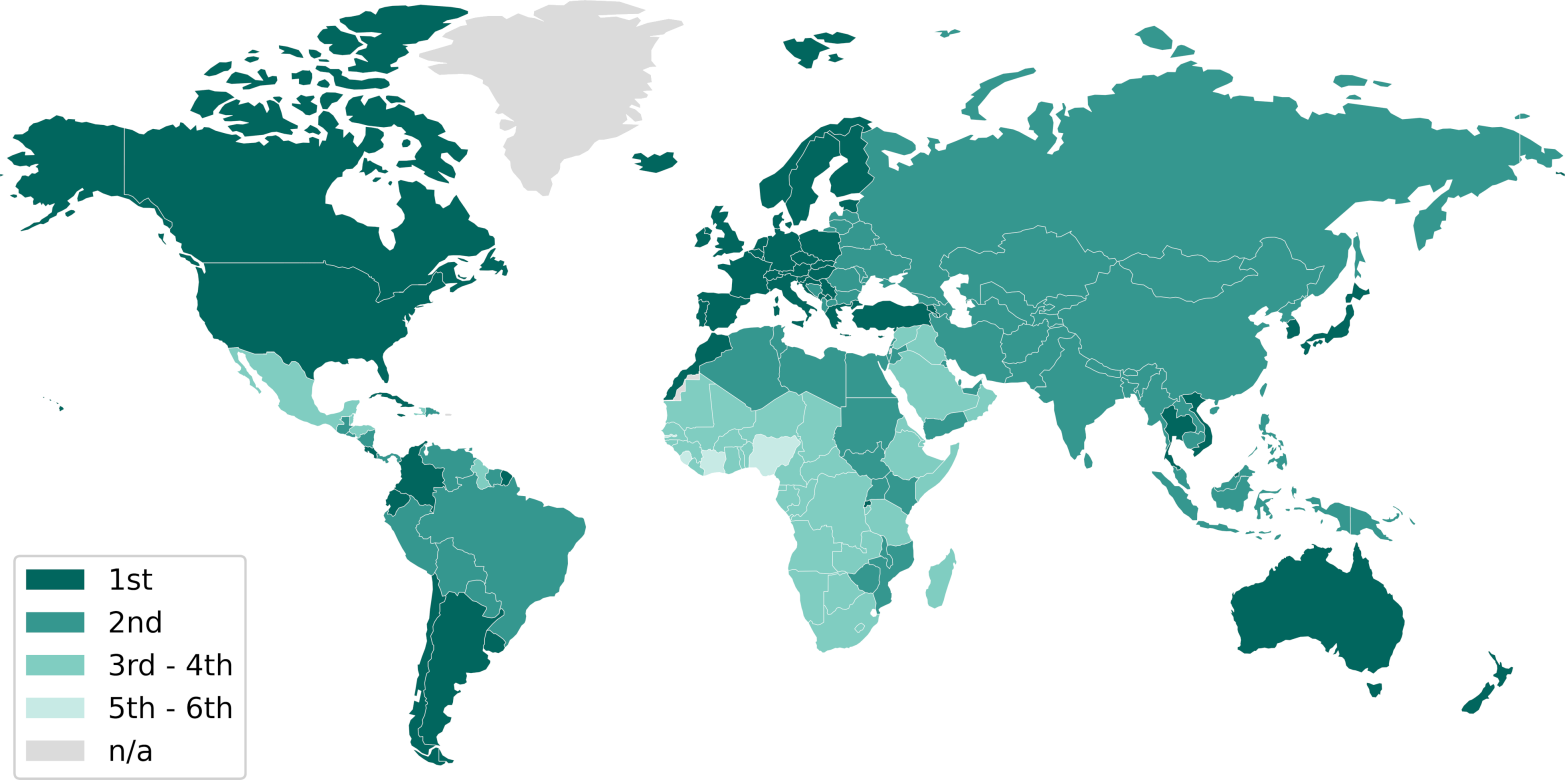
Ranking of cancer as the cause of death in 2016 for the population below 70 years old (premature mortality). Data sourced from the WHO World Cancer Report [20].

Despite our understanding of preventable contributing factors, cancer remains a global burden, affecting over 10 million people annually. As of 2016, it is the leading cause of death in 55 countries (Figure 2), particularly those with high Human Development Index (HDI) values. The higher cancer burden in high-HDI countries is attributed to the development of vaccines and treatments for infectious diseases and other causes of premature mortality [20–21]. With a 53% drop in cardiovascular disease mortality rates in countries with very high HDI between 1981–1985 and 2006–2010, the cancer mortality rate decreased by only 17%. The decrease is even lower (5%) in countries with medium-to-high HDI. The inadequacy of prevention strategies and the persistent prevalence of cancer emphasise the importance of developing appropriate and effective anticancer therapies so that the global community can work towards reducing the overall burden of cancer.

In 2000, Douglas Hanahan and Robert Weinberg introduced the hallmarks of cancer [22], a comprehensive set of capabilities and characteristics that define the cellular and molecular traits of cancer cells. These hallmarks have been updated every decade [23–24]. The hallmarks framework has enabled researchers to develop more nuanced, spatially informed approaches to cancer therapy, reinforcing its role in driving multidisciplinary strategies for predicting treatment response [25].

Targeted drug delivery is an emerging, nontraditional approach in cancer treatment and an active research area. In this approach, engineered NMs, acting as nanocarriers, selectively and specifically target cancer cells to deliver drug payloads. The NMs can distinguish between cancer cells and healthy cells; thereby minimising adverse effects. Moreover, these nanocarriers can bypass cancer cell resistance mechanisms, thereby enhancing overall drug efficacy. Targeted drug delivery aims at overcoming the side effects and limitations of traditional cancer treatment, like surgery, radiotherapy, and chemotherapy. Surgery, though effective in some cases, is invasive and not always feasible, especially if cancer has metastasised. Radiation therapy similarly suffers if the cancer has metastasised, and may damage healthy tissues and organs, causing long-term complications. Chemotherapy can also indiscriminately kill healthy cells and result in adverse side effects, while also losing efficacy as cancer cells develop resistance. Within chemotherapy, various drug delivery methods exist, each with its advantages and limitations (refer to Supporting Information File 1, Table S1). The choice depends on the patients’ needs and the type of cancer treated.

## Perspective

**Figure 3:**
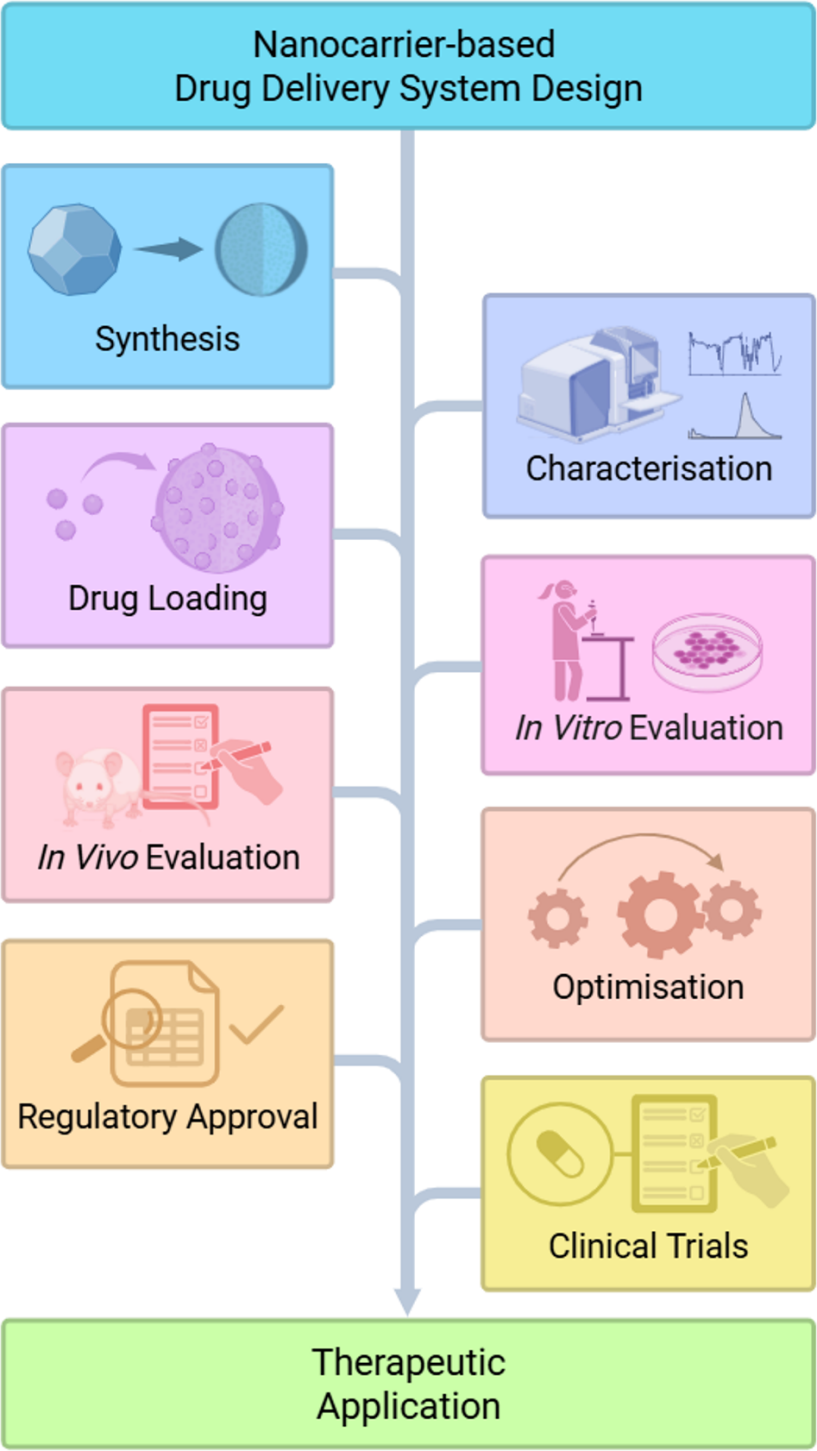
A generalised schematic overview of the development pathway for a carbon nanoparticle-based drug delivery system. Created in BioRender. Bartkowski, M. (2025) https://BioRender.com/goba3a2. This content is not subject to CC BY 4.0.

The development of a novel CNM-based drug delivery system for targeted anticancer therapeutic delivery is a complex and multistep process that involves several key milestones (Figure 3), each of which requires distinct expertise in materials science, chemistry, biology, and regulatory affairs. Achieving these key milestones is critical to developing a safe and effective drug delivery system that can improve the treatment of cancer.

The process begins with the synthesis of CNMs tailored to specific size and surface chemistry requirements, using methods such as chemical vapour deposition, laser ablation, or electrochemical techniques, among others. These CNMs must then be thoroughly characterised to confirm their structural and physicochemical properties, including size, shape, charge, surface functionality, stability, and potential toxicity. Following characterisation, anticancer therapeutics are loaded onto the CNMs via approaches like physical adsorption, covalent attachment, or encapsulation. The resulting formulations undergo in vitro evaluation, and the promising candidates then proceed to in vivo testing in animal models to evaluate therapeutic efficacy, biodistribution, and safety. Insights from these studies guide further optimisation of the delivery system, which may involve adjusting particle size, surface chemistry, or drug-loading strategies. Once optimised and evaluated, the system enters regulatory review, requiring preclinical safety and efficacy data to meet approval criteria for human trials. The final stage involves clinical trials across multiple phases to validate the system’s safety and effectiveness in treating cancer, ultimately determining its suitability for clinical use.

### Challenges associated with the production of CNMs

The large-scale production of CNMs for drug delivery presents several practical and economic challenges. These include high equipment and raw material costs, limited yields, quality control issues, and environmental and safety concerns. Addressing these barriers requires targeted innovation in synthesis methods, improved standardisation, and interdisciplinary collaboration. An overview of key production challenges is provided in Table 1.

**Table 1:** Overview of challenges associated with the production of CNMs for drug delivery purposes.

Challenge	Overview

the need for specialised equipment	The production of CNMs requires specialised, often costly equipment such as high-temperature furnaces or chemical reactors, and careful maintenance to ensure quality. While this is not unique to nanomedicine, as traditional pharmaceutical manufacturing also relies on specialised tools, the required expertise is distinct because the instrumentation used for CNMs differs significantly from that used for small molecule drugs.
raw material costs	The raw materials used to produce CNMs can be expensive; however, other targeted delivery systems, such as viral vectors, often involve even higher costs. For example, carbon nanotubes are typically produced using high-purity graphite, which can be expensive and difficult to obtain. To reduce raw material costs, one solution is to use alternative or more cost-effective raw materials. For example, using biomass-derived carbon precursors [26] or recycled materials can reduce costs and improve the sustainability of the production process.
yield limitations/scalability	The yield of CNMs from a given production process can be low, which can limit their practical applications. For example, some methods for producing carbon nanotubes have a yield of only a few per cent, which can make them too expensive for large-scale applications. To increase yields, one solution is to optimise the production process to reduce waste and maximise the conversion of raw materials to CNMs. This can involve improving the catalysts used in the production process or optimising the reaction conditions to reduce unwanted side reactions. Moreover, scalability can be addressed by employing production methods that are easily scalable, such as using continuous flow reactors [27].
quality control issues	The production of CNMs can be complex, and small variations in the production process can affect their properties and performance. Quality control is, therefore, critical in ensuring the production of high-quality CNMs, but this can add to the time and expense of production. To address quality control issues, one solution is to develop new methods for characterising and quantifying CNMs during the production process. This may involve advanced characterisation techniques, including microscopy and spectroscopy, to monitor CNM size, shape, and purity in real-time or post-synthesis.
environmental concerns	The production of CNMs can generate waste and consume significant energy, contributing to environmental burden. One possible solution is to develop more sustainable production methods, such as using renewable energy sources or recycling waste materials [26].
contamination	CNMs can carry impurities or residual chemicals from the production process, which can affect their properties and performance. Therefore, it is crucial to implement quality control measures that can detect and remove such contaminants.
safety concerns	The production of CNMs can pose safety risks to workers, as the materials can be inhaled [28] or come into contact with skin. To address this issue, it is important to implement appropriate safety protocols and provide proper training and protective equipment for workers.

The lack of standardised methods for the synthesis and characterisation of CNMs is a major challenge in the field of nanotechnology. CNMs, such as carbon nanotubes and graphene, have unique properties that make them attractive for a wide range of applications, including drug delivery, electronics, and energy storage [29]. However, the lack of standardised methods for their synthesis and characterisation can complicate their regulatory approval and limit their practical applications.

One of the main issues is the wide variety of methods that can be used to synthesise CNMs, which can lead to variations in their properties and performance. For example, carbon nanotubes can be synthesised using arc discharge, laser ablation, and chemical vapour deposition, among others [30]. Each method can result in carbon nanotubes with different structures, sizes, and surface chemistries, which can affect their biological activity, toxicity, and efficacy as drug delivery vehicles.

Similarly, the characterisation of CNMs can be challenging due to their small size and complex structures. However, there is a lack of standardised methods for measuring these properties, which can make it difficult to compare results across studies and to ensure the safety and efficacy of CNM-based drug delivery systems.

Efforts are underway to address these issues and to establish standardised methods for the synthesis and characterisation of CNMs. For example, organisations such as the National Institute of Standards and Technology and the International Organisation for Standardisation (ISO) are working to develop consensus-based standards for the characterisation of nanomaterials [31–33]. These efforts aim to improve the reproducibility and comparability of results across studies and to facilitate the regulatory approval of carbon nanomaterial-based drug delivery systems.

### Model therapeutics in nanocarrier development

Chemotherapy drugs are designed to kill rapidly dividing cancer cells by disrupting their cell division and DNA synthesis. However, they can also affect healthy cells in the body that divide rapidly, such as cells in the hair follicles, bone marrow, digestive system, and reproductive system. This can lead to a range of side effects, some of which can be severe.

Take the chemotherapeutic agent doxorubicin (DOX), for example. Used in cancer treatment for decades, DOX has become a mainstay in oncology and is often nicknamed “the red devil” [34]. This name stems not only from its distinctive red colour, attributed to the quinone ring in its chemical structure, which absorbs in the visible spectrum and makes the drug clearly visible during intravenous administration, but also from its high potency and the wide range of severe side effects it can cause.

These side effects include myelosuppression, where reduced blood cell production in the bone marrow can lead to anaemia, bruising, and higher infection risk; cardiotoxicity, which may result in heart failure or complications, especially with higher cumulative doses or existing heart conditions; severe nausea and vomiting if not managed with antiemetics; hair loss, which can be temporary or permanent; mucositis, involving inflammation of the mouth, throat, and gastrointestinal tract, causing pain and diarrhoea; skin reactions such as rash and redness; and a higher risk of secondary cancers, including leukaemia.

Most of anticancer therapeutics are also generally associated with flu-like symptoms, such as fever, chills, and muscle aches, and in rare cases, allergic reactions, which may include hives, difficulty breathing, and swelling of the face, lips, tongue, or throat. The severity of the side effects may vary depending on the dose, duration of treatment, and individual patient factors; and so, can be managed with supportive care and medications, and the dose and duration of treatment can be tailored to minimise toxicity.

The use of nanocarriers for the delivery of anticancer therapeutics is a promising strategy for improving the efficacy of the drug while minimising its toxicity to healthy tissues. This approach has the potential to reduce the incidence and severity of side effects associated with traditional chemotherapy (Table 2).

**Table 2:** Key limitations of conventional chemotherapy and how nanocarrier-based delivery can improve patient outcomes.

Chemotherapy alone	With nanocarrier delivery

**lack of specificity** damages both cancerous and healthy cells	**targeted delivery** selectively accumulates in cancer cells, sparing healthy tissue
**dose-dependent toxicity** higher doses cause more severe side effects	**controlled release** maintains steady drug levels, reducing peak toxicity
**poor solubility** limits efficacy and may increase toxicity	**enhanced solubility** improves bioavailability and therapeutic performance
**rapid clearance** short half-life necessitates frequent or high dosing	**slower clearance** extends drug action, enabling lower and less frequent dosing
**variable patient response** metabolism and clearance rates differ widely	**greater consistency** patient-tailored properties improve individual outcomes

There are several model anticancer therapeutics that are commonly used in nanocarrier design, such as doxorubicin or gemcitabine. By encapsulating or loading these therapeutics onto nanocarriers, researchers can improve their pharmacokinetics and pharmacodynamics, enhance their therapeutic efficacy, and reduce their toxicity [35–36]. This allows for a metric of comparison between the efficacy of drug-only delivery and nanocarrier-mediated delivery.

When selecting a model cargo to support the design of a novel drug delivery system (DDS), it is important to take its clinical properties into account. For instance, gemcitabine is a widely used chemotherapeutic agent and a clinically relevant model cargo in nanocarrier design, owing to its established role in the treatment of pancreatic, lung, bladder, and breast cancers. Its small molecular weight and good aqueous solubility facilitate encapsulation in diverse DDSs, while its instability and short half-life, due to rapid deamination and phosphorylation, highlight the need for protective nanocarriers to improve bioavailability. These features make gemcitabine ideal for standardised evaluation of novel DDSs, enabling direct comparison with conventional formulations.

### Evaluation of CNMs for nanoformulations

Different classes of CNMs can exhibit markedly distinct biological behaviours despite their shared carbon framework. Variations in dimensionality, aspect ratio, surface area, and surface chemistry result in unique biodistribution patterns, metabolic fates, and pharmacokinetic profiles. For instance, the high aspect ratio of carbon nanotubes (CNTs) contributes to their prolonged pulmonary retention, often linked to chronic inflammation and fibrotic responses [37]. In contrast, a recent first-in-human investigation of thin, highly purified graphene oxide nanosheets reported that acute inhalation was well tolerated, showing no adverse effects on lung function, cardiovascular health, or systemic inflammatory markers [28].

Even within the same material class, substantial variability in structure and surface chemistry can arise depending on the synthesis and purification methods employed, resulting in divergent biological outcomes. For example, carbon dots (CDs) smaller than 6 nm are typically cleared rapidly via the renal route, whereas larger CDs tend to evade renal filtration due to the formation of a protein corona that effectively increases their hydrodynamic size [38]. Beyond renal clearance, particle size also critically influences toxicity, metabolic fate, tumour targeting, protein interactions, and hepatic processing. Additional physicochemical attributes, such as surface charge, further modulate the overall biological profile of CNMs, underscoring the complex interplay between nanoscale properties and biological behaviour [39].

Moreover, post-synthesis surface modifications can substantially diversify the physicochemical properties of a given CNM, leading to distinct biological behaviours. For instance, diethylentriaminepentaacetic dianhydride-functionalised multiwalled CNTs exhibited markedly different biodistribution profiles following intravenous administration in mice, depending on their degree of functionalisation. CNTs with a low degree of functionalisation accumulated in liver, spleen, and lungs, whereas highly functionalised CNTs accumulated in bladder, suggesting different elimination pathways [40]. Overall, the intrinsic and engineered variability of CNMs underscores the need for rigorous physicochemical characterisation coupled with systematic biological evaluation to ensure reliable assessment of safety and performance.

### Physicochemical evaluation of CNMs

Size and shape are critical parameters that influence the biological behaviour of CNMs. These factors affect biodistribution, clearance, and cellular uptake. For instance, small carbon nanoparticles (CNPs) are readily cleared through renal pathways, whereas larger or irregularly shaped CNPs tend to accumulate in organs such as the liver and spleen. Characterisation of these properties is typically performed using dynamic light scattering, electron microscopy, or atomic force microscopy.

Surface charge plays a significant role in determining how CNMs interact with biological membranes and intracellular environments. It influences processes such as cellular uptake, cytotoxicity, and inflammatory signalling. For example, BSA-derived negatively charged CDs exhibited enhanced cellular uptake, whereas positively charged CDs elicited stronger cytokine responses [41]. Zeta potential measurements are routinely used to quantify surface charge, which can be tuned through appropriate surface functionalisation strategies.

Stability is another essential aspect of NM evaluation. CNMs must retain their structural and functional integrity under physiological conditions, including varying pH, ionic strength, and temperature. Instability can result in aggregation, degradation, or loss of therapeutic payload. Strategies such as lyophilisation, drug encapsulation, and the use of stabilising surface coatings are employed to preserve nanoparticle stability over time.

The degree of functionalisation is also a key consideration as surface modifications can enhance solubility, targeting ability, and biocompatibility. Consistent functionalisation is necessary to ensure reproducibility and to maintain the desired therapeutic and safety profiles. Assessing this aspect includes quantifying the density and distribution of attached ligands or therapeutic agents.

In addition to evaluating the NMs themselves, the manufacturing process must be carefully monitored. Reproducibility, scalability, and material purity are essential for translational development. Contaminants such as carboxylated carbonaceous fragments (CCFs) may be introduced during synthesis and processing, and these impurities can alter biological responses or induce toxicity. Their detection and removal should form a routine part of quality assurance protocols.

### Biological evaluation of CNMs

Biological evaluation begins with in vitro assays to assess cellular viability, compatibility with blood components, and cellular uptake. These studies provide early insights into the safety and performance of CNM-based formulations. This is followed by in vivo testing, which offers a more complete understanding of pharmacokinetics, biodistribution, therapeutic efficacy, and systemic toxicity. Animal studies help identify accumulation patterns, potential off-target effects, and immune responses that are not evident in simpler biological models.

Determining the appropriate dosage is another crucial factor as therapeutic performance is influenced by drug loading capacity, drug release kinetics, and loading efficiency. Careful optimisation of these parameters is required to achieve effective drug delivery while minimising harm to healthy tissues.

Toxicity remains one of the most significant concerns associated with CNMs. Their unique physical and chemical properties, while advantageous for drug delivery, can also contribute to harmful effects. Potential mechanisms of toxicity include the generation of reactive oxygen species, disruption of cellular membranes, prolonged retention in tissues, and activation of immune responses. These risks highlight the importance of careful nanoparticle design and thorough preclinical evaluation.

Biodegradability represents another crucial factor governing the clinical translation of CNMs. Persistent materials raise concerns regarding long-term accumulation and potential toxicity, whereas those susceptible to enzymatic or oxidative degradation offer safer clearance pathways. Recent studies have shown that CNOs can undergo biodegradation upon exposure to human myeloperoxidase, horseradish peroxidase, or UV-assisted photo-Fenton processes. Notably, oxidised CNOs degrade more readily than their pristine counterparts, due to their higher defect density and greater abundance of oxygen-containing functional groups [42]. These findings highlight that the biodegradability of CNMs strongly depends on their surface chemistry and structure, emphasizing the need for systematic evaluation of their degradation pathways and by-products to ensure the safe design of nanomedicines.

Moreover, CNMs can contain impurities that may impact their biological activity and safety. Therefore, it is important to assess the purity of the NMs and remove any impurities before their use, such as CCFs [43]. Studies have shown that CCFs can induce oxidative stress and inflammation in cells, potentially leading to cellular damage and toxicity [44]. The potential toxicity of CCFs is a concern in the field of CNM research, as they can be difficult to remove during purification and processing. Ongoing efforts are focused on developing methods to detect and quantify CCFs in CNM products and to better understand their potential toxicity.

The biocompatibility of CNMs engineered as DDSs requires thorough, layered evaluation. Depending on the intended route of delivery and application, this may involve cytotoxicity assays to assess effects on cell viability and metabolism, and hemocompatibility assays to evaluate interactions with blood components such as red blood cells, clotting factors, and platelets. In vitro release studies provide insight into drug release kinetics based on NM properties and formulation. Animal toxicity studies help determine in vivo safety profiles across organ systems, while biodistribution and pharmacokinetic studies elucidate how CNMs are metabolised, cleared, and accumulate in the body, guiding safe and effective dosing strategies.

Taken together, the physicochemical and biological assessment of CNMs forms the foundation for their responsible development and clinical use. Establishing standardised methods for these evaluations will be essential to ensure consistency, regulatory compliance, and patient safety.

### Shortcomings in standardisation

#### Lack of standardised evaluation

Standardising the evaluation of CNM-based nanocarriers for targeted anticancer drug delivery remains a major barrier to clinical translation. The diversity of CNM structures, variability in drug-loading strategies, and complexity of cancer biology all contribute to inconsistencies in preclinical testing. Without harmonised evaluation protocols, it is difficult to compare findings across studies or draw reliable conclusions about therapeutic performance and safety (Table 3).

**Table 3:** Challenges in standardising the evaluation of carbon nanomaterial-based nanocarriers for targeted anticancer therapeutic delivery.

Challenge	Description

lack of established protocols	No universally accepted protocols exist for evaluating CNM-based nanocarriers, making it difficult to compare safety and efficacy across studies.
complex interactions and cancer heterogeneity	CNMs interact unpredictably with biological systems, while cancer itself varies by type and stage, complicating standardised assessment.
difficulty in defining suitable controls and targets	To establish the efficacy of CNM-based nanocarriers for targeted anticancer therapeutic delivery, suitable controls need to be included in the evaluation protocols. However, finding appropriate controls for comparison can be challenging, particularly for nanocarriers that are designed to target specific cellular receptors or pathways. Appropriate control groups need to be identified to ensure the accuracy of the results and establish the efficacy of the nanocarrier in comparison to existing treatments.
reproducibility of results	Variability in nanocarrier design, formulation, and testing conditions often leads to inconsistent results across laboratories.
safety considerations	There are concerns about the potential toxicity and long-term effects of CNMs on the body. Standardised evaluation protocols need to include appropriate safety assessments, including biocompatibility, cytotoxicity, and immunotoxicity assays, to ensure the safety of these nanocarriers for use in humans.

Addressing the lack of standardised evaluation is essential to realise the clinical potential of CNM-based DDSs. Key priorities include developing robust protocols for assessing efficacy, reproducibility, and safety; such protocols are ideally tailored to account for nanocarrier-specific properties and cancer heterogeneity. The standardisation process requires systematic assessment, quantification, and reporting of CNM properties, including size, shape, stability, surface charge and functionalisation, as well as payload density and distribution. Once production and characterisation procedures are standardised, the preclinical evaluation of carbon-based DDS should follow the same criteria applied to small bioactive agents, specifically assessing CNM toxicity in the selected model, accumulation rates within cancer tissue, and therapeutic efficacy. To achieve this, techniques such as immunohistochemical analysis of model organs or the use of radiolabelled DDSs are essential. Ultimately, coordinated efforts among researchers, regulators, and industry will be critical to establishing consistent benchmarks that support the reliable comparison, optimisation, and approval of nanocarrier-based therapies.

#### Lack of standardised nomenclature

The term nanomaterial has been defined in the EU’s Recommendation on the Definition of a Nanomaterial (originally defined in 2011/696/EU, and updated in 2022/C 229/01) [45]. In regulating the definition, it was intended to capture the unique properties and potential risks associated with materials at the nanoscale. This was highly successful, and the EU established a common understanding and regulatory framework for the safe use of nanotechnology in various industries.

Presently, a major challenge facing CNPs, and nanomaterials overall, is the need to refine and standardise their classification and nomenclature. Currently, it is difficult to compare results across studies, which can lead to confusion and inconsistencies in scientific literature. The present situation is due to the diversity of nanomaterials and the challenges associated with accurately characterising and measuring their properties, as well as the rapid pace of discovery and innovation in the field. For instance, CDs are not well-defined due to a high variation in structural composition, the nomenclature of which is a current point of debate [46]. Moreover, there is a lack of publicly available, centralised databases on nanocarriers, leading researchers to rely heavily on review articles that quickly become outdated, cannot be maintained, and are often non-comprehensive.

Efforts are underway to improve the current standards in nomenclature and classification; several organisations, such as the ISO and the American Society for Testing and Materials, have developed guidelines and standards for the characterisation and measurement of nanomaterials, including definitions of key terms and concepts (Table 4). However, these guidelines are not comprehensive. Refining and universally adopting them is essential to foster the stratification of CNMs based on their physicochemical and functional properties, thereby establishing a common classification toward standardization. Consequently, standardising the classification and nomenclature of CNMs, as well as developing relevant databases, will be essential for advancing research and development in this field, improving the safety and efficacy of CNM formulations, and enabling regulatory approval and translation.

**Table 4:** Selected international standards and guidelines relevant to the characterisation, nomenclature, and evaluation of nanomaterials.

Standard	Scope / Description

ISO 80004-1:2023	This document defines key terms in nanotechnology, aiming to enhance communication between industry professionals and their collaborators [31].
ASTM E2834-12	This standard provides guidance for the measurement of particle size distribution in nanomaterials using dynamic light scattering and transmission electron microscopy (TEM) techniques [47].
ISO/TR 10993-22:2017	This standard provides a framework for the biological evaluation of medical devices composed of or containing nanomaterials, as well as those generating nano-objects through degradation or mechanical processes. Overall, this standard addresses material characterisation, sample preparation, risk assessment, and common challenges in testing nanomaterials compared to bulk materials [32].
ISO/TS 11888:2017	This standard outlines methods for characterising mesoscopic shape factors of multiwalled carbon nanotubes, using techniques such as SEM, TEM, viscometry, and light scattering. It includes measurement methods for static bending persistence length, drawing on concepts from polymer physics to define these properties [33].

#### Lack of standardised regulation

**Figure 4:**
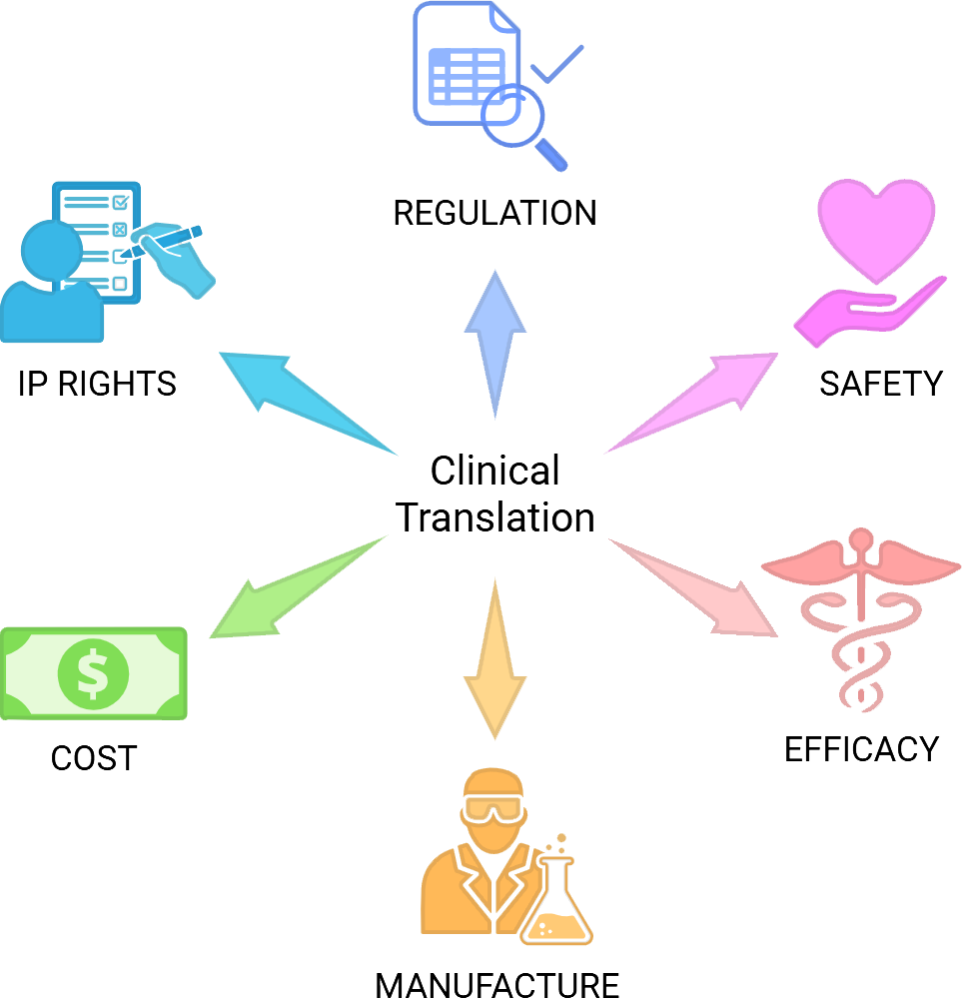
A selection of key challenges and opportunities associated with the clinical translation of a CNP-based DDS. Created in BioRender. Bartkowski, M. (2025) https://BioRender.com/goba3a2. This content is not subject to CC BY 4.0.

Translating preclinical drug delivery research to the clinic is a complex and lengthy process that is associated with several interlinked challenges, as well as opportunities [48]. These include regulatory hurdles, safety and efficacy concerns, manufacturing scalability, cost-effectiveness, and intellectual property considerations (Figure 4). Regulatory approval processes are often lengthy and vary by country, requiring early engagement with agencies to ensure alignment. Ensuring patient safety demands comprehensive preclinical studies evaluating toxicity and pharmacological profiles. Demonstrating efficacy similarly requires well-designed animal studies to assess therapeutic outcomes. Scalable and reproducible manufacturing is essential for clinical translation, while cost considerations necessitate the use of efficient, low-cost materials and methods. Finally, navigating intellectual property rights and licencing can be complex, underscoring the value of collaboration with legal and commercial partners.

The regulatory landscape for CNMs is complex, uncertain, and continuously evolving, reflecting efforts to assess their potential risks and benefits. Oversight varies across regions and involves multiple agencies (Table 5). In the EU, the European Chemicals Agency and the European Medicines Agency govern the regulation of CNMs. In the UK, the Health and Safety Executive and the UK REACH framework play key roles. In the US, regulation is led by the Environmental Protection Agency and the Food and Drug Administration. For additional perspective, Japan’s Ministry of Economy, Trade and Industry has also developed guidelines for nanomaterials. These agencies work globally to ensure the safe development and use of CNMs. Together, such agencies contribute to the safe development and use of CNMs worldwide.

**Table 5:** Summary of select regulatory frameworks across USA, EU, UK and Japan governing nanoscale materials, highlighting key agencies, legislative instruments, and guidance initiatives.

Region	Regulatory Overview

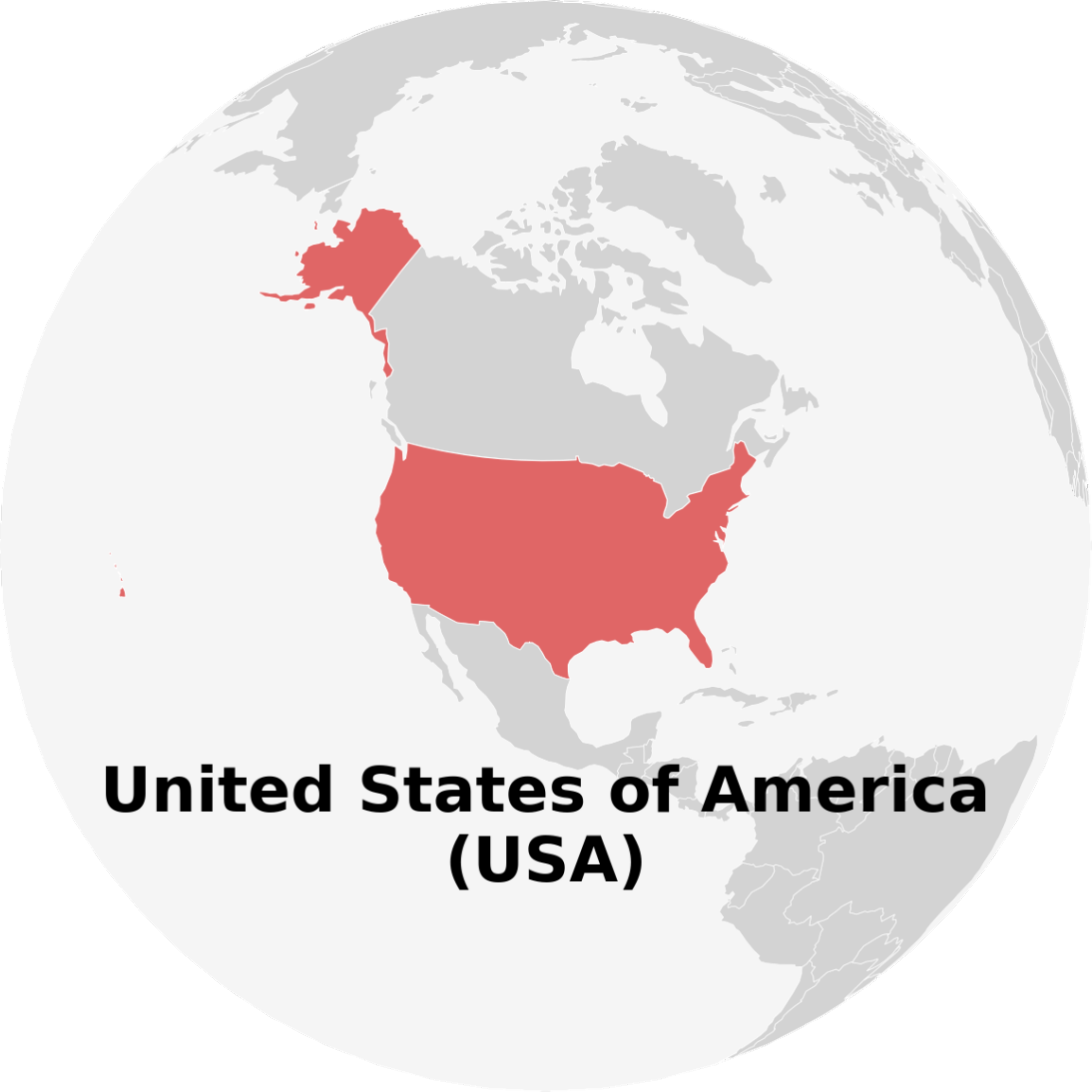	In the United States, the Environmental Protection Agency (EPA) initiated the Nanoscale Materials Stewardship Program (NMSP) in 2008–2009 to encourage companies to voluntarily provide information on the manufacture, use, and exposure of nanomaterials [49]. Currently, the EPA regulates nanomaterials under the Toxic Substances Control Act (TSCA), requiring manufacturers and importers to report specific information about nanoscale materials [50]. The Food and Drug Administration (FDA) has issued guidance documents for the development of nanotechnology-based products, including drug products. These documents provide recommendations on the safety, effectiveness, and quality of such products [51]. The FDA continues to work on streamlining the regulatory process for nanotechnology-based products to ensure their safe and effective use [52].
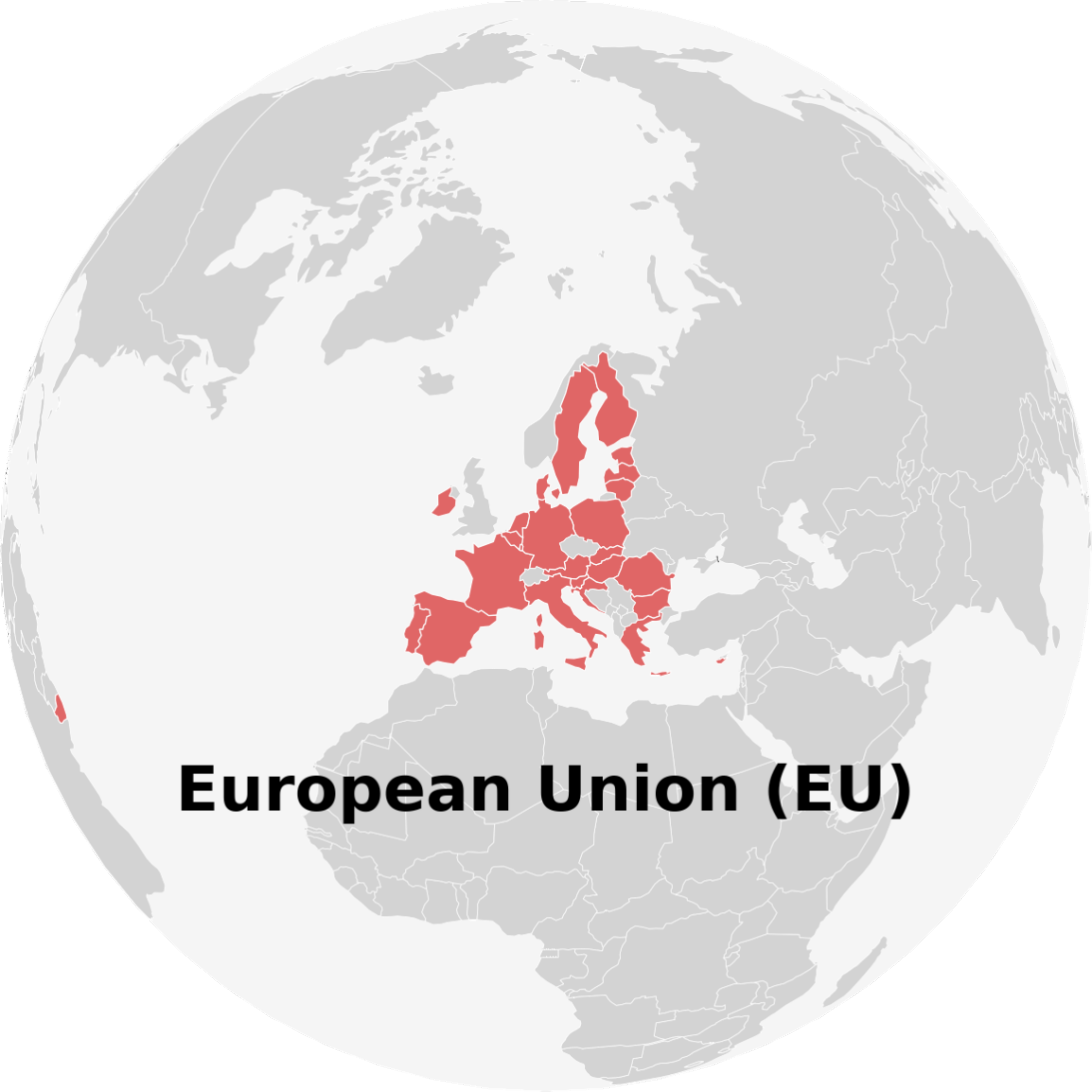	In the EU, the regulatory landscape for CNMs is governed by the European Chemicals Agency (ECHA) under the Registration, Evaluation, Authorisation and Restriction of Chemicals (REACH) regulation. REACH includes specific provisions for nanomaterials, requiring manufacturers and importers to register their substances with ECHA, providing detailed data on properties, uses, toxicology, environmental impact, exposure, and risks. ECHA evaluates these submissions to determine if further actions, such as restrictions, labelling, or additional testing, are necessary, while responsibility for safety remains with manufacturers. This approach ensures compliance, promotes safe use, and supports innovation. Additional EU regulations are established for specific industries, governing the use of nanomaterials in food, cosmetics, and other consumer products. For instance, the Cosmetics Regulation (EC No 1223/2009) [53] mandates labelling and safety assessments for cosmetics containing nanomaterials before market entry.
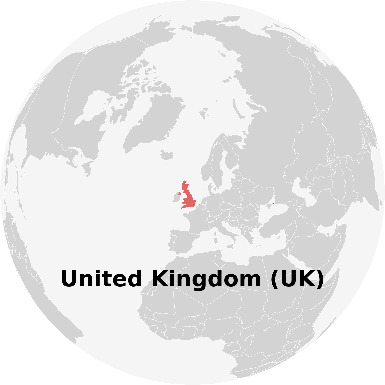	In the UK, regulatory oversight of nanomaterials is primarily handled by the Health and Safety Executive (HSE), which enforces workplace health and safety regulations, including the Control of Substances Hazardous to Health Regulations 2002. The HSE provides guidelines for the safe handling and use of nanomaterials, with a focus on risk assessment, exposure control, and management. Following Brexit, the UK has established its own regulatory framework, including the UK REACH [54], which largely mirrors EU REACH but operates independently. This framework governs the registration, evaluation, and risk management of nanomaterials to ensure their safe use within the UK.
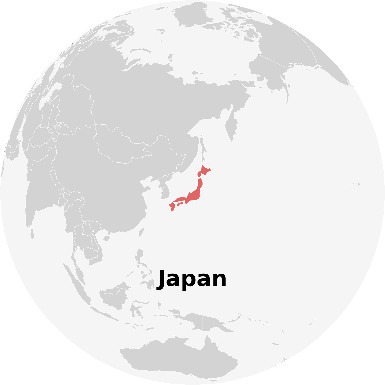	In Japan, the Ministry of Economy, Trade and Industry has issued guidelines for the safe handling and management of nanomaterials [55]. These guidelines focus on risk assessment, exposure control, and workplace safety, supporting industries in implementing effective risk management strategies for nanomaterials. They also promote voluntary information sharing along the supply chain and encourage manufacturers to adopt proactive safety measures in cooperation with relevant ministries.

The complexity of the regulatory landscape reflects not only the diverse applications of CNMs but also their transformative potential across industries. Regulatory bodies are actively working to develop and refine frameworks, such as REACH and TSCA, as well as others, to address the unique properties and challenges posed by CNMs. Collaborative efforts are underway to establish standardised testing protocols and safety evaluation methods to ensure the responsible development of these materials, supporting their use in innovative applications like drug delivery, while safeguarding health and the environment.

## Conclusion

CNMs undeniably represent a promising frontier in drug delivery, offering a unique combination of high surface area, tuneable properties, and demonstrated biocompatibility. As explored in this perspective, their potential to revolutionise therapeutic delivery, particularly in addressing the global burden of cancer, is substantial. However, to achieve this, a collaborative effort is needed to overcome the existing challenges.

Translating CNM-based nanocarriers from conception to clinic demands a rigorous and standardised approach across all stages of development. Key considerations highlighted include the need for robust and reproducible synthesis and characterisation methods to ensure consistency and minimise batch-to-batch variations. Furthermore, a comprehensive understanding of their interaction with biological systems, encompassing both physicochemical and biological evaluations, is needed to mitigate potential toxicity and optimise therapeutic efficacy. The current shortcomings in standardisation, spanning nomenclature, evaluation methodologies, and regulatory frameworks, present significant hurdles that must be overcome through international collaboration and the establishment of clear guidelines.

Despite these complexities, the ongoing research focused on reducing CNM toxicity, refining synthesis protocols, and establishing clear regulatory pathways offers promising perspectives. The acceleration of CNM translation hinges on the continued development of new models for therapeutic delivery, the integration of advanced characterisation techniques, and the establishment of globally harmonised protocols and regulations facilitated by the international dedicated agencies. Ultimately, by addressing these critical considerations and fostering a collaborative environment, the immense promise of CNMs as nanocarriers can be fully realised, paving the way for innovative and highly effective therapeutic interventions in cancer and beyond.

## Supporting Information

File 1 Overview of conventional drug delivery methods in cancer therapy, including administration routes, key characteristics, and example drugs.

## Data Availability

All data that supports the findings of this study is available in the published article and/or the supporting information of this article.
