# Supplementary material for: Toward clinical translation of carbon nanomaterials in anticancer drug delivery: the need for standardisation
Source: Beilstein J Nanotechnol. 2025 Nov 18;16:2092–104. doi: 10.3762/bjnano.16.144 (PMC12642948; doi:10.3762/bjnano.16.144)
Supplement: File 1 — Overview of conventional drug delivery methods in cancer therapy, including administration routes, key characteristics, and example drugs. [file Beilstein_J_Nanotechnol-16-2092-s001.pdf]

BEILSTEIN JOURNAL OF NANOTECHNOLOGY

Supporting Information

for

Toward clinical translation of carbon nanomaterials in anticancer drug delivery: the need for standardisation

Michał Bartkowski, Francesco Calzaferri and Silvia Giordani

Beilstein J. Nanotechnol. **2025,** *16,* 2092–2104. doi:10.3762/bjnano.16.144

Overview of conventional drug delivery methods in cancer therapy, including administration routes, key characteristics, and example drugs

License and Terms: This is a supporting information file under the terms of the Creative Commons Attribution License (https://creativecommons.org/licenses/by/4.0). Please note that the reuse, redistribution and reproduction in particular requires that the author(s) and source are credited and that individual graphics may be subject to special legal provisions.
The license is subject to the *Beilstein Journal of Nanotechnology* terms and conditions: (https://www.beilstein-journals.org/bjnano/terms)

Table S1: Overview of conventional drug delivery methods in cancer therapy, including administration routes, key characteristics, and example drugs.

Drug Delivery Method	Overview	Example Drugs
**Intravenous (IV) Injection**	Drugs are injected directly into the bloodstream through a vein. The advantage of this method is that the drugs can quickly reach the entire body, including the cancer cells. However, IV injection is associated with systemic toxicity and can cause side effects such as nausea, vomiting, and fatigue.	paclitaxel (Taxol)<br>*breast, lung, and ovarian cancers*<br><br>cisplatin (Platinol)<br>*lung, bladder, and ovarian cancers*<br><br>doxorubicin (Adriamycin)<br>*breast, bladder, and ovarian cancers*
**Oral Delivery**	Drugs are taken by mouth in the form of capsules or tablets. The advantage of oral administration is that it is a convenient and non-invasive method. However, oral administration is associated with lower drug bioavailability, which can limit the effectiveness of the treatment.	tamoxifen (Nolvadex)<br>*hormone receptor-positive breast cancer*<br><br>imatinib (Gleevec)<br>*chronic myeloid leukaemia and some gastrointestinal cancers*
**Localised Delivery**	Drugs are delivered directly to the tumour or affected area using techniques like intratumoral injection, catheterisation, or implants. This can reduce side effects linked to systemic delivery but may be invasive and limited by tumour size or location.	Pegylated liposomal doxorubicin (Doxil)<br>*ovarian and breast cancers*<br>*[liposomal delivery]*<br><br>5-Fluorouracil (5-FU)<br>*colorectal, breast, and other*<br>*[sustained-release implants]*
**Transdermal Delivery**	Drugs are delivered through the skin using a patch or gel. The advantage of transdermal delivery is that it is non-invasive and can provide sustained drug release. However, transdermal delivery is associated with low drug bioavailability, which can limit the effectiveness of the treatment.	Fentanyl (Duragesic)<br>*pain associated with cancer through a transdermal patch*<br><br>Scopolamine (Transderm Scop)<br>*nausea associated with cancer chemotherapy through a transdermal patch*
